# Long-Term Molecular Remission after Treatment with Imatinib in a Chronic Myeloid Leukemia Patient with Extreme Thrombocytosis Harboring Rare e14a3 (b3a3) BCR::ABL1 Transcript: A Case Report

**DOI:** 10.3390/curroncol29110645

**Published:** 2022-10-28

**Authors:** Xupai Zhang, Haoping Sun, Yi Su, Hai Yi

**Affiliations:** Department of Hematology, The General Hospital of Western Theater Command, Chengdu 610083, China; xupaizhanglego@163.com (X.Z.); 32374906@qq.com (H.S.); suhang1234@hotmail.com (Y.S.)

**Keywords:** chronic myeloid leukemia, imatinib, extreme thrombocytosis, e14a3 (b3a3) BCR::ABL1 transcript

## Abstract

An atypical BCR::ABL1 fusion gene transcript in chronic myeloid leukemia (CML) patients, even those with variant Philadelphia (Ph) chromosome translocation, is very rare. In the present study, we report a case of CML (41 years, female) with extreme thrombocytosis at onset, with the variant Ph chromosome and rare e14a3 (b3a3) BCR::ABL1 transcript. The patient was prescribed imatinib as a first-line therapy and subsequently achieved complete hematologic remission within 2 months and major molecular response (MMR) within 3 months, and the transcript was undetectable within half a year. During up to nine years of follow-up, the quantification of this rare fusion gene was consistently negative with no BCR::ABL1 kinase domain mutations. Furthermore, we collected previously reported CML cases with the e14a3 (b3a3) transcript that indicated that the e14a3 (b3a3) transcripts appeared to have a larger number of thrombocytosis and variant Ph translocations than CML in general. This subgroup of CML might have better responses and outcomes to imatinib than patients with common transcripts.

## 1. Introduction

Chronic myeloid leukemia (CML) is one of the myeloproliferative neoplasms (MPNs) originating from acquired malignant clones of pluripotent hematopoietic stem cells and characterized by remarkable leukocytosis in peripheral blood, in which immature granulocytes, basophilia and eosinophilia are predominant. As is well known, its signature feature is the formation of the Philadelphia (Ph) chromosome, a result of a reciprocal translocation, t (9; 22) (q34; q11), leading to genetic fusion between the breakpoint cluster region (BCR) on 22q11 and Abelson 1 (ABL1) on 9q34 [1,2]. The BCR::ABL1 transcript encodes the corresponding oncoprotein, which possesses abnormal constitutive tyrosine kinase activity and plays a huge role in the occurrence of CML [3]. Marked leukocytosis and splenomegaly are the particularly outstanding characteristics of CML, and approximately fifty percent of patients discover thrombocytosis, but usually no more than 1000 × 10^9^/L, at initial diagnosis [4,5]. In general, ninety-five percent of newly diagnosed CML patients have Ph chromosomes detected by means of conventional karyotype analysis; nevertheless, one or more additional chromosomes are added to the Ph chromosome in some patients, called variant Ph chromosome translocation [6,7]. In terms of BCR::ABL1 transcripts in CML, the most common breakpoints of the BCR gene are located in BCR introns downstream of exon 13 or 14 (M-BCR), while a few involve exon 1 or 19 (m-BCR or u-BCR, respectively), and the breakpoint of the ABL1 gene is usually located in introns downstream of ABL1 exon 2. The resultant fusion transcripts are of the following three subtypes: (i) e13a2 (b2a2) or e14a2 (b3a2), translated into a 210 kDa protein termed p210; (ii) e1a2, translated into a 190 kDa protein termed p190; (iii) e19a2, translated into a 230 kDa protein termed p230 [3,8]. However, less than one percent of patients have breakpoints of the BCR or ABL1 gene at other locations, thus forming atypical BCR::ABL1 fusion gene transcripts [9,10].

To date, studies focused on the classical Ph chromosome or common BCR::ABL1 transcripts in CML have been comprehensive and thorough. Nevertheless, the diagnosis, treatment and prognosis of cases with atypical BCR::ABL1 transcripts, including those with an additional variant translocation, have received relatively little attention. In the present study, we report a case of a CML patient with extreme thrombocytosis, a variant Ph chromosome and a rare e14a3 (b3a3) BCR::ABL1 transcript who achieved long-term remission at the molecular level after imatinib (Glivec) treatment during up to nine years of follow-up.

## 2. Case Presentation

A 41-year-old female patient with no dizziness, fatigue, abdominal distension, bruising or thrombus presented to the Department of Hematology after being noted to have thrombocytosis in June 2013. One year prior, this patient was found to have mildly elevated platelets during a routine physical examination, but no treatment was given because she had no symptoms. She had no previous medical or surgical history or any relevant hematological family history. However, a complete blood count (CBC) analysis performed a few days before admission displayed an extremely elevated platelet count of 1742 × 10^9^/L and a slightly increased leukocyte count of 12.12 × 10^9^/L, which was confirmed by a second CBC (1703 × 10^9^/L and 11.00 × 10^9^/L, respectively). Therefore, she was referred to our inpatient ward for further examination and treatment.

**Figure 1:**
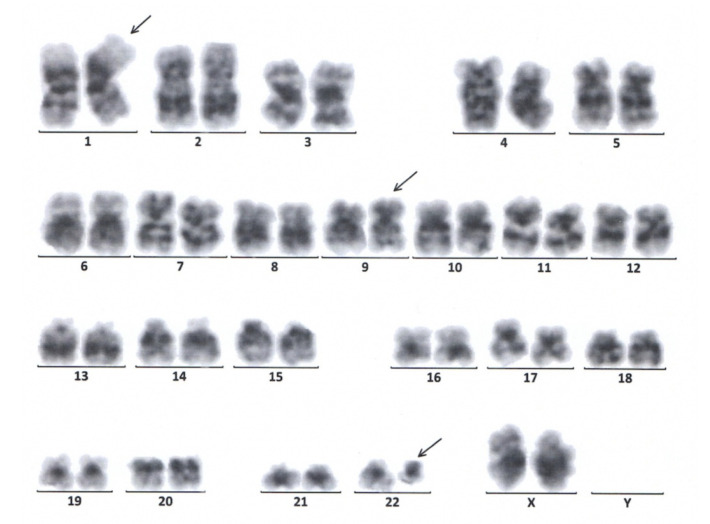
The G-banded karyotype indicated the t (1; 9; 22) (p36.3; q34; q11) translocation.

**Figure 2:**
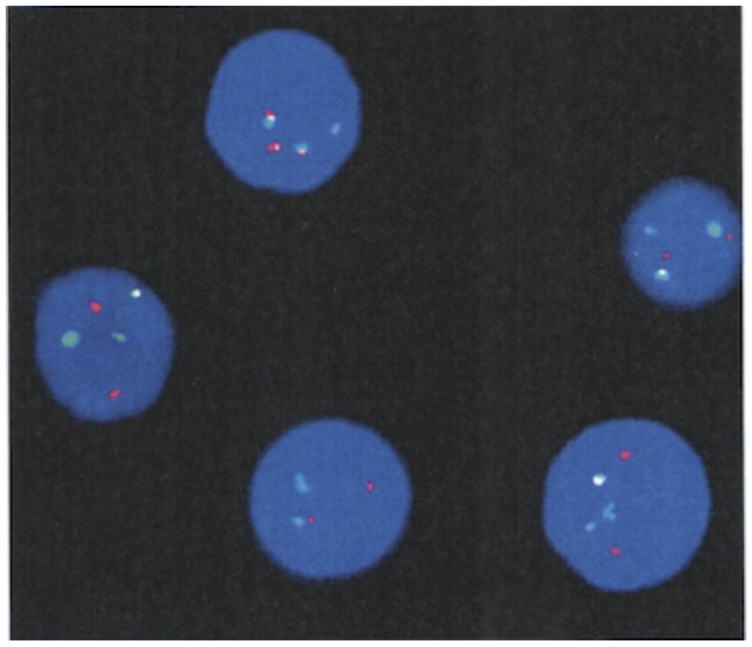
The dual-color and dual-fusion FISH of the BCR::ABL1 fusion gene was 1F2R2G (82%), 2F1R1G (10%) and 2R2G (8%) (the red signal (R) indicates the ABL1 probe; the green signal (G) indicates the BCR probe; F indicates the fusion signal; normal nucleus, 2R2G; nucleus with a classical t (9,22) translocation, 2F1R1G; cut-off value, 1F (11%), 2F (2%)).

A physical examination performed in our hospital revealed no abnormalities. In particular, no hepatomegaly or splenomegaly was detected either by palpation or ultrasound. Her clinical characteristics and blood tests from a sample drawn at the time of admission are described in Table 1. A bone marrow smear revealed that granulocyte proliferation was obviously active and accounted for 61.6% of the karyocytes, among which immature granulocytes were predominant, with 13% eosinophils, 8.2% basophils and 2.2% blasts; megakaryocytes were easy to see, some of which were dwarf in morphology, and no large or giant granulocytes were discovered. A bone marrow biopsy displayed 81–100% cellularity with granulocytic predominance and evident megakaryocytic hyperplasia; the reticulin stain failed to discover increased reticulin fibrosis; the possibility of essential thrombocythemia (ET) was considered. Molecular biological examination detected no JAK2 mutations (p. 523_547 and p. 599_621; V617F and exon 12), CALR mutations (p. 352_418) or MPL mutations (p. 501_521; S505 and W515), which excluded the diagnostic signature genes of ET. The results of commercial reverse transcription polymerase chain reaction (RT-PCR) kits for the detection of common BCR::ABL1 transcripts were negative, including e13a2 (b2a2), e14a2 (b3a2), e1a2 and e19a2. The conventional karyotype analysis revealed an atypical t (1; 9; 22) (p36.3; q34; q11), a variant Ph chromosome, in all the analyzed cells (Figure 1). Fluorescence in situ hybridization (FISH) with a BCR::ABL1 dual-color dual-fusion probe kit indicated that the BCR::ABL1 fusion gene was positive in 92% (276/300) of the interphase cells examined, the specific signal patterns of which were 1F2R2G (82%), 2F1R1G (10%) and 2R2G (8%) (the red signal (R) indicates the ABL1 probe; the green signal (G) indicates the BCR probe; F indicates the fusion signal) (Figure 2). Meanwhile, subsequent nested polymerase chain reaction (nested PCR) combined with agarose gel electrophoresis designed to detect rare BCR::ABL1 transcripts revealed a subtype of e14a3 (b3a3). Additionally, type-specific real-time quantitative polymerase chain reaction (qPCR) was performed with the designed primers (forward primer, CACGTTCCTGATCTCCTCTGAC; reverse primer, ACACCATTCCCCATTGTGATTAT), and the expression of the e14a3 (b3a3) BCR::ABL1 transcript was detected using cDNA synthesis. The ratio of the e14a3 (b3a3) BCR::ABL1 transcript was 84.05% (the copy number of target gene BCR::ABL1 was 84454; the copy number of control gene ABL1 was 100,480; the measure of sensitivity was 1.00 × 10^−5^) [9,10]. Hence, a diagnosis of chronic-phase CML was ultimately confirmed.

At the time of the initial diagnosis, the patient was subjected to interferon α-2b, hydroxyurea and aspirin for nearly half a month and plateletpheresis twice to decrease the platelet count and produce antithrombosis. However, the effect of decreasing the platelet count appeared to be unsatisfactory. When the diagnosis was verified, imatinib (Glivec, 400 mg daily) was prescribed as a targeted therapy for CML accompanied by aspirin (100 mg daily). Blood routine examination, the quantification of the e14a3 (b3a3) BCR::ABL1 transcript and BCR::ABL1 kinase domain mutations were regularly performed in the outpatient department, and the molecular response was evaluated every 3 months until major molecular response (MMR) was achieved and then evaluated every 3, 6 or 12 months. During treatment, the patient did experience neutropenia related to the therapy and had to reduce the dose of imatinib. This patient achieved complete hematologic remission within 2 months and MMR within 3 months, and the transcript was undetectable within half a year. To date, during up to nine years of follow-up, the quantification of this rare fusion gene was consistently negative with no BCR::ABL1 kinase domain mutations.

## 3. Discussion

As mentioned above, approximately fifty percent of CML cases have thrombocytosis at initial diagnosis. The platelet count of a few patients can even exceed 1000 × 10^9^/L, and the degree of increase has no significant correlation with the leukocyte count. We are supposed to differentiate CML from other MPNs on the basis of the Ph chromosome or BCR::ABL1 fusion gene, especially ET, although concomitant clonal abnormalities rarely exist in CML and other MPNs [11,12]. A persistent platelet count >1000 × 10^9^/L is one of the diagnostic criteria for accelerated-phase CML, which is rare in chronic-phase CML [13]. In our study, the patient initially had an extremely elevated platelet count and a slightly raised leukocyte count, and a bone marrow biopsy suggested the possibility of ET. However, the positive BCR::ABL1 fusion gene and negative JAK2, CARL and MPL mutations accompanied by eosinophilia and basophilia excluded the possibility of ET. In addition, previously reported studies showed that the bone marrow of CML with extreme thrombocytosis generally shows dwarf megakaryocytes with round nuclei, while ET shows mature, large or giant megakaryocytes [5,14]. The majority of CML patients with marked thrombocytosis are female [15,16], and this group of patients seldom experiences thrombotic and hemorrhagic complications [15,17]. Other studies indicated that hydroxyurea cannot decrease the platelet count when awaiting confirmation of the diagnosis of CML [4]. Once a tyrosine kinase inhibitor (TKI) is administered, the platelet count quickly drops to normal [4,18]. These phenomena were consistent with our case.

**Figure 3:**
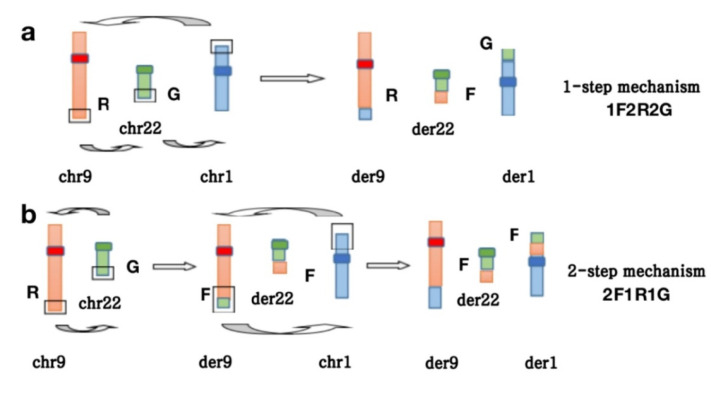
(**a**,**b**). FISH determines the mechanism of the formation of the t (1; 9; 22) (p36.3; q34; q11) translocation (R indicates the ABL1 gene; G indicates the BCR gene; F indicates the fusion gene; chr indicates chromosome; der indicates derivative chromosome). (**a**) One-step mechanism (1F2R2G: 1 fusion signal on der22, 2 red signals on der9 and normal chr9, 2 green signals on der1 and normal chr22). (**b**) Two -step mechanism (2F1R1G: 2 fusion signals on der22 and der1, 1 red signal on normal chr9, 1 green signal on normal chr22).

One or more additional chromosomes are added to the Ph chromosome, called a variant Ph translocation, which is found in approximately five percent of newly diagnosed CML patients [7]. It can be formed between the translocation of 22q11 and another chromosome other than chromosome 9 or among the complex translocations of 9, 22 and other chromosomes. All chromosomes except chromosome Y can be involved in variant Ph translocations. Regardless of the classical and variant Ph chromosomes, the recombination of 9q34 and 22q11 is fundamental in the formation of the Ph chromosome, which can be detected using FISH or molecular biological methods. Dual-color and dual-fusion FISH is an available technique to detect the mechanism of the formation of variant translocations. At present, there are two different mechanisms (1-step and 2-step mechanisms) that can generate a variant Ph translocation involving one chromosome being added to chromosomes 9 and 22. The former is a chromosomal translocation that simultaneously occurs among three different chromosomes with a 3-break event, and the latter involves a sequential translocation of the third chromosome into the classical Ph chromosome with a 4-break event [6,7,19]. We can determine the translocation pattern of the variant Ph chromosome according to the FISH signal patterns. In the present study, conventional karyotype analysis revealed an atypical t (1; 9; 22) (p36.3; q34; q11) in all of the analyzed cells, and the specific signal patterns of the BCR::ABL1 fusion gene were 1F2R2G (82%), 2F1R1G (10%) and 2R2G (8%). Therefore, we concluded that the variant Ph chromosomes of this patient was formed mostly via the 1-step mechanism, and a few were formed by the 2-step mechanism (Figure 3). A previous study reported three cases with variants of the Ph chromosome involving chromosomes 1, 9 and 22 that were treated with imatinib. Two of them failed to reach complete cytogenetic remission, and one achieved complete cytogenetic remission within a year [7]. However, some reported studies clarified that regardless of the mechanism and how many chromosomes are involved, the Ph chromosome variant has no influence on the patient’s response to TKI or the prognosis of CML [7,20].

Moreover, the BCR::ABL1 transcripts in a very few CML patients are not among the three common subtypes (p210, p190 and p230), thus forming atypical BCR::ABL1 transcripts. Among these, the breakpoints of the BCR gene can be located outside the three well-defined regions and even exit the inserted sequence, while the breakpoints of the ABL1 gene arise in introns downstream of ABL1 exon 3. More than ten atypical BCR::ABL1 transcripts have been detected in CML patients, such as e13a3 (b2a3), e14a3 (b3a3), e13e14a3, e1a3, e4a2, e6a2, e8a2, e12a2, e16a2 and e18a2 [9,10,21]. Among them, the prevalence of the e14a3 (b3a3) BCR::ABL1 transcript is fairly low, ranging from 0.21% to 0.3% [9,10,22], and the number of focused studies is relatively low due to these being infrequent cases. To clarify the clinical characteristics and outcomes of CML patients with the e14a3 (b3a3) BCR::ABL1 fusion gene transcript, we conducted a systemic literature search on PubMed and Embase, using keywords and MeSH terms for chronic myeloid leukemia, CML, e14a3 and b3a3. In addition, we also reviewed the references of the retrieved literature. Currently, 62 cases with e14a3 (b3a3) transcripts were reported, including our present patient, of which 25 cases had relatively detailed information of the clinical characteristics and treatments (Table 2) [22,23,24,25,26,27,28,29]. Notably, male patients accounted for 84% (21/25) of the CML cases with the e14a3 (b3a3) transcript, which was consistent with previous studies [1]. The median age of these patients was 48 years (range: 19–83 years), while their leukocyte and platelet counts were 45 × 10^9^/L and 546.5 × 10^9^/L (range: 9–300 and 207–1375, respectively). Approximately 78% (14/18, some cases lacked data about platelet counts) of the patients had thrombocytosis, defined as a platelet count >300 × 10^9^/L, which was higher than the 50% in general CML patients [15]. Of the 25 cases, 20% (5/25) of the patients had variant Ph translocations, the frequency of which was significantly higher than CML in general, as described previously (20% vs. 5%) [7]. Eleven patients (cases 1–3, case 5, case 7, cases 12–16 and case 19) were treated with imatinib as a first-line therapy and achieved good remission, although the treatment of two patients (case 13 and case 19) subsequently switched to dasatinib because of the E255K mutation and poor tolerance, respectively, and one patient (case 16) relapsed after complete cytogenetic remission and died shortly after. In addition, some studies elucidated that the patients with e14a3 (b3a3) transcripts have better responses and outcomes to imatinib than patients with common transcripts [9,10]. For some reason, the e14a3 (b3a3) transcript lacks exon 2, which encodes part of the Src homology domain 3 (SH3) region. The deletion of the SH3 region appears to increase the activity of TKI, and simultaneously, its loss might reduce the activation of the STAT5 signaling pathway and weaken leukemogenesis, resulting in a good clinical course [22,30].

## 4. Conclusions

In conclusion, e14a3 (b3a3) BCR::ABL1 transcripts, even with additional variant translocation, are rare in CML. We can confirm the possible translocation pattern of the variant Ph chromosome by means of FISH. The e14a3 (b3a3) transcripts appear to have a larger number of thrombocytosis and variant Ph translocations than CML in general, and these patients might have better responses and outcomes to imatinib than patients with common transcripts. Further studies of a larger number of CML patients with e14a3 (b3a3) transcripts should be implemented to verify these findings.

## Figures and Tables

**Table 1:** The clinical characteristics of this patient at diagnosis.

Complete Blood Count	
Leukocytes (×10^9^/L)	10.5
Neutrophils (%)	69.0
Eosinophils (%)	3.00
Basophils (%)	5.00
Lymphocytes (%)	20.0
Monocytes (%)	2.00
Erythrocytes (×10^12^/L)	4.44
Hemoglobin (g/L)	120
Platelets (×10^9^/L)	1375
Relative risk	
Sokal	1.16 (intermediate)
EURO	328 (low)
EUTOS	35 (low)

**Table 2:** The characteristics of 25 CML patients with e14a3 (b3a3) BCR::ABL1 transcripts.

Case No.	Age/ Sex	Stage	Leukocyte (×10^9^/L)	Platelet (×10^9^/L)	Chromosome Karyotype	Treatment	Follow-Up
1	41/F	CP	10.5	1375	t (1; 9; 22)	Hu + IFN-α; followed by IM	MR within 6 months
2	66/M	CP	36.4	1045	t (9; 22; 11)	Hu; followed by IM	MR within 4 months
3	57/M	CP	61	1017	t (9; 22; 12)	Hu; followed by IM	MR within 3 months
4	52/M	CP	53.91	207	t (9; 22)	NIL	CCyR within 4 months
5	67/M	CP	48.1	252	t (9; 22)	Hu; followed by IM	MR within 12 months
6	54/F	CP	13	1209	t (9; 22)	Hu; followed by NIL	NA
7	40/M	CP	46.42	275	t (9; 22)	Hu; followed by IM	response well *
8	83/M	CP	NA	NA	t (8; 9; 22)	NA	NA
9	44/M	AP	NA	NA	t (9; 22)	NA	NA
10	49/M	BP	NA	NA	t (9; 22)	NA	MR within 9 months
11	22/M	CP	NA	NA	t (9; 22)	NA	NA
12	24/M	CP	222	938	t (9; 22)	Hu; followed by IM	response well *
13	52/M	CP	229	590	t (9; 22)	Hu + IFN-α; IM; followed by DAS	E255K mutation
14	41/M	AP	26	414	t (9; 22)	Hu; followed by IM	MR within 6 months
15	41/M	CP	115	798	t (9; 22)	IFN-α; followed by IM	MR within 6 months
16	48/F	CP	300	435	t (9; 22)	Hu + IFN-α; IM; followed by VP	MR within 3 months
17	48/M	CP	98.2	1072	t (9; 22)	Hu; followed by HSCT	CCyR within 4 months
18	30/M	CP	45	NA	t (9; 22)	NA	NA
19	81/M	CP	28	NA	t (9; 22)	IM; DAS; followed by Hu	PCyR within 1 month
20	69/M	CP	29.9	286	t (9; 22)	IFN-α	NA
21	69/M	CP	18	527	t (4; 9; 22)	Hu; busulfan; followed by 6-MP	NA
22	51/M	CP	19.9	566	t (9; 22)	IFN-α; followed by HSCT	NA
23	23/M	CP	95	485	t (9; 22)	Hu + IFN-α	NA
24	19/M	CP	42	381	t (9; 22)	Hu + IFN-α	NA
25	39/F	CP	9	NA	t (9; 22)	NA	NA

M, male; F, female; CP, chronic phase; AP, accelerated phase; BP, blast phase; Hu, hydroxyurea; IFN-α, interferon α; V, vincristine; P, prednisone; IM, imatinib; DAS, dasatinib; NIL, nilotinib; 6-MP, 6-mercapthopurine; HSCT, hematopoietic stem cell transplantation; NA, not available; CCyR, complete cytogenetic response; PCyR, partial cytogenetic response; MR, molecular response (undetectable BCR::ABL1 transcript); *, the details were not given.

## Data Availability

The data supporting the conclusions of this article are included within the article. More details are available upon request.
